# Optimizing C-TIRADS for sub-centimeter thyroid nodules using machine learning–derived feature importance

**DOI:** 10.3389/fendo.2025.1668347

**Published:** 2025-09-26

**Authors:** Dongming Guo, Zhihui Lin, Jiajia Wang, Xianying Liao, Haiqing Huang, Yuxia Zhai, Zhe Chen

**Affiliations:** ^1^ Department of Interventional Ultrasound, Cancer Hospital of Shantou University Medical College, Shantou, China; ^2^ Department of Ultrasound, Cancer Hospital of Shantou University Medical College, Shantou, China; ^3^ Department of Ultrasound, Second Affiliated Hospital of Shantou University Medical College, Shantou, China

**Keywords:** sub-centimeter thyroid nodules, C-TIRADS, machine learning, SHAP, ultrasound, risk stratification, microcarcinoma

## Abstract

**Background:**

To optimize the diagnostic performance of the Chinese Thyroid Imaging Reporting and Data System (C-TIRADS) for sub-centimeter thyroid nodules by incorporating machine learning–derived feature importance.

**Methods:**

This retrospective study included 741 patients in a primary cohort and 421 patients in an external validation cohort. SHapley Additive exPlanations (SHAP) were used to quantify the diagnostic contribution of six ultrasound features based on an XGBoost model. A modified C-TIRADS scoring system was developed by assigning greater weight to the most contributive feature while retaining original weights for other features. Diagnostic performance was evaluated using the area under the receiver operating characteristic curve (AUC), net reclassification improvement (NRI), and decision curve analysis (DCA).

**Results:**

SHAP analysis identified vertical orientation as the most predictive feature for malignancy in sub-centimeter nodules. The modified scoring system significantly improved diagnostic performance in both the primary (AUC: 0.911 vs. 0.898, P < 0.001) and validation cohorts (AUC: 0.931 vs. 0.899, P < 0.001). NRI analysis further showed a substantial improvement in risk classifications, with NRI values of 0.406 in the primary and 0.471 in the validation cohort (both P < 0.001). DCA demonstrated greater net clinical benefit across wider threshold ranges in both cohorts. Additionally, malignancy rates exhibited a more rational stepwise increase from C-TIRADS 4A to 5, indicating improved risk stratification.

**Conclusion:**

The SHAP-guided modified C-TIRADS scoring system enhances diagnostic accuracy and risk stratification for sub-centimeter thyroid nodules and may facilitate improved clinical decision-making in this challenging subset.

## 1 Introduction

Thyroid nodules are common findings in the general population and represent one of the most prevalent endocrine disorders. With the widespread use of high-resolution ultrasonography, the detection rate of thyroid nodules has increased significantly (1). Approximately 25% to 68% of the global population harbors thyroid nodules, the majority of which are benign (2–4). However, 5% to 15% of these nodules are malignant (5, 6). Therefore, accurate risk stratification is crucial to avoid unnecessary invasive surgeries and missed diagnoses of thyroid cancer. This is particularly important for sub-centimeter nodules (≤10 mm in maximum diameter), where the limited spatial resolution of ultrasound often results in ambiguous sonographic features and ongoing controversy regarding the indications for fine-needle aspiration (7, 8).

The Chinese Thyroid Imaging Reporting and Data System (C-TIRADS) is a structured ultrasound-based risk stratification tool and widely adopted in China (9). It assigns risk scores based on a combination of suspicious sonographic features, such as composition, echogenicity, shape, margin, and calcification, to guide clinical management. While C-TIRADS has demonstrated good diagnostic performance overall, emerging evidence (10–12) suggests that the diagnostic utility of positive ultrasound features may differ depending on nodule size, particularly for thyroid nodules ≤ 10 mm, for which all positive features have low diagnostic efficacy. These findings raise the possibility that a size-adjusted scoring strategy, which accounts for the differential predictive value of specific ultrasound features, may enhance the diagnostic accuracy of C-TIRADS. However, this concept remains insufficiently investigated in the current literature.

In this study, we aim to evaluate the relative diagnostic contributions of individual C-TIRADS ultrasound features for sub-centimeter thyroid nodules using a machine learning-based predictive model. We further propose a modified C-TIRADS scoring system that adjusted feature weights for nodules ≤ 10 mm, and assess whether this revision improves diagnostic efficacy compared to the original C-TIRADS system.

## 2 Methods

### 2.1 Study population and nodule selection

This retrospective study consisted of a primary cohort and an external validation cohort. The primary cohort comprised 741 patients from the Second Affiliated Hospital of Shantou University Medical College between January 2019 and December 2024, while the validation cohort included 421 patients form Cancer Hospital of Shantou University Medical College between June 2020 and May 2025. All patients underwent either ultrasound-guided fine-needle aspiration or surgical resection, with a definitive pathological diagnosis. Some patients had multiple benign nodules multifocal papillary thyroid carcinoma; to ensure accurate correspondence between the target nodule and the pathological diagnosis, only one representative nodule (the largest or most suspicious) per patient was included in the analysis. All included nodules were evaluated using grayscale ultrasound prior to pathological confirmation.

All included cases had complete demographic information, sonographic reports containing all required ultrasound features for C-TIRADS scoring, and definitive diagnostic outcomes based on fine-needle aspiration biopsy or surgical pathology. The inclusion criteria were as follows: (1) availability of high-quality preoperative ultrasound images; (2) complete documentation of C-TIRADS-related ultrasound features; (3) a definitive diagnosis based on cytology or histopathology. The exclusion criteria were as follows: (1) Nodules presenting with purely cystic composition or a classic spongiform appearance; (2) incomplete imaging or clinical data; (3) poor-quality ultrasound images not permitting accurate feature assessment or target nodule identification; (4) nodules with ambiguous or indeterminate diagnostic outcomes; (5) history of previous neck radiation or any antitumor treatment prior to thyroid nodule diagnosis.

### 2.2 Ultrasound feature extraction and definitions

All preoperative ultrasound examinations were performed using high-frequency linear array probes on two main ultrasound systems: the Mindray Resona 7 (Mindray, Shenzhen, China) and the GE LOGIQ E9 (GE Healthcare, Chicago, IL, USA). All nodules were re-evaluated using the stored ultrasound images. Two sonographers with 5–10 years of experience in thyroid ultrasound independently assessed image quality and, when the retained images were clear and standardized, performed feature extraction according to the 2020 C-TIRADS criteria (9). In cases of disagreement, a third senior sonographer with more than 20 years of experience adjudicated to reach consensus. Structured data were then generated based on this standardized process, and these data were used for subsequent model training.

To ensure standardization, reproducibility, and comparability across cases, we restricted the analysis to the six core ultrasound features explicitly defined by the 2020 C-TIRADS. The evaluated features included composition, echogenicity, shape, margin, calcification, and artifacts. Nodule composition was categorized as solid (entirely or nearly entirely composed of soft tissue), predominantly solid (solid portion >50% of the nodule volume), and predominantly cystic (cystic or fluid-filled portion >50% of the nodule volume). Echogenicity was classified as iso/hyperechoic (echogenicity equal to or greater than the surrounding thyroid parenchyma), hypoechoic (lower echogenicity than the thyroid parenchyma but higher than or equal to the strap muscles), and markedly hypoechoic (echogenicity lower than the adjacent neck strap muscles). Shape was assessed by the vertical orientation, defined as an anteroposterior diameter greater than the transverse diameter on transverse imaging. Margin characteristics were recorded as ill-defined or irregular when the nodule boundaries appeared blurred, spiculated, or uneven. Calcifications were further subtyped as microcalcifications (punctate echogenic foci ≤1 mm without acoustic shadowing), macrocalcifications (coarse calcifications >1 mm with posterior shadowing), or peripheral calcifications (calcifications located along the rim of the nodule). Extrathyroidal extension was defined as the disruption of the thyroid capsule, capsular bulging, or direct invasion of surrounding structures. Comet-tail artifact was defined as a short, bright, tapering reverberation artifact extending posteriorly from echogenic spots within the nodule.

### 2.3 C-TIRADS scoring and risk stratification

According to the 2020 C-TIRADS guideline, each ultrasound feature is assigned a score: one point is given for each suspicious feature, including solid composition, markedly hypoechoic echogenicity, vertical orientation, ill-defined or irregular margin or extrathyroidal extension, and microcalcification. A comet-tail artifact, when not accompanied by microcalcification, is considered a benign feature and is assigned −1 point. The total score is then used to stratify the nodule into one of six C-TIRADS categories: C-TIRADS 2 (−1 point), C-TIRADS 3 (0 point), C-TIRADS 4A (1 point), C-TIRADS 4B (2 points), C-TIRADS 4C (3–4 points) and C-TIRADS 5 (≥5 points).

### 2.4 Machine learning and SHAP analysis

An eXtreme Gradient Boosting (XGBoost) model was applied to comprehensively assess the diagnostic contribution of six key ultrasound features derived from the C-TIRADS guideline in primary cohort: vertical orientation, solid composition, markedly hypoechoic echogenicity, microcalcification, ill-defined/irregular margin or extrathyroidal extension, and comet-tail artifact (counted only when not coexisting with microcalcifications). SHapley Additive exPlanations (SHAP) values were calculated using the ExactExplainer algorithm to quantify the contribution of each feature to the model’s prediction.

### 2.5 Modified scoring system construction and validation

Based on the findings that the most impactful features in primary cohort, a modified C-TIRADS scoring system was proposed by increasing the weight of the most contributive feature. In the modified scoring system, this top-contributing feature was assigned a weight of 2 points, while the remaining features retained their original weight of 1 point.

To compare the diagnostic performance between the original and modified C-TIRADS scoring systems, receiver operating characteristic (ROC) curve analysis was performed, and the area under the ROC curve (AUC) was calculated for each system. The statistical significance of AUC differences was assessed using the DeLong test. Reclassification performance between scoring systems was compared using the net reclassification improvement (NRI), and 95% confidence intervals (CIs) and corresponding P-values for the NRI were calculated using 1,000 bootstrap iterations. Decision curve analysis (DCA) was conducted to quantify the net clinical benefit of each scoring model across a range of threshold probabilities.

Additionally, the modified scoring system was externally validated in the validation cohort using the same analytic procedures described above.

### 2.6 Statistical analysis

Continuous variables were summarized as mean ± standard deviation or median with interquartile range depending on distribution. Categorical variables were presented as frequencies and percentages. Group comparisons of categorical variables were performed using the chi-square test or Fisher’s exact test as appropriate. Comparisons of continuous variables were conducted using the independent samples t-test or Mann–Whitney U test.

All analyses were performed using Python (version 3.12.2) and R (version 4.4.3). A two-sided *P*-value < 0.05 was considered statistically significant.

## 3 Results

### 3.1 Patient characteristics

**Figure 1:**
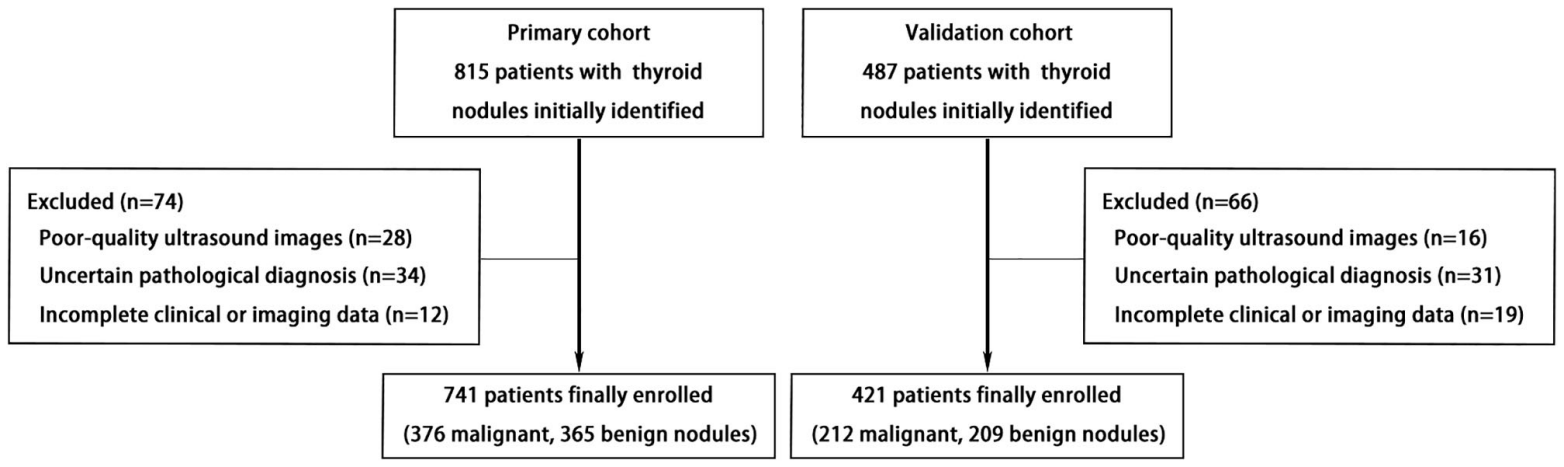
Flowchart of patient enrollment and selection in the primary and validation cohorts.

A flowchart illustrating the patient selection and exclusion process is presented in **Figure 1**. The baseline characteristics of the two cohorts are summarized in **Table 1**. No significant differences were observed in age or sex distribution between the primary and validation cohorts. Most ultrasound features showed no statistically significant differences between the two cohorts, except for the following: margins, solid composition, markedly hypoechoic echogenicity, peripheral calcifications. The overall malignancy rates were comparable between the two cohorts. **Table 2** presents the distribution of C-TIRADS ultrasound features among the malignant cases.

**Table 1:** Baseline characteristics of patients in the primary and validation cohorts.

Characteristics	Primary cohort n=741 (%)	Validation cohort n=421 (%)	*P*
Sex			0.518
Male	132 (17.8)	68 (16.2)	
Female	609 (82.2)	353 (83.8)	
Age (years)	48 (39-56)	47 (34-59)	0.164
Orientation			0.236
Parallel	458 (61.8)	245 (58.2)	
Vertical	283 (38.2)	176 (41.8)	
Margin			
Circumscribed	530 (71.5)	255 (60.6)	< 0.001
Ill-defined or irregular	180 (24.3)	158 (37.5)	< 0.001
Extrathyroidal extension	60 (8.1)	15 (3.6)	0.003
Composition			
Solid	634 (85.6)	341 (81.0)	0.046
Predominantly solid	89 (12.0)	64 (15.2)	0.126
Predominantly cystic	18 (2.4)	16 (3.8)	0.206
Echogenicity			
Iso/hyperechoic	179 (24.1)	123 (29.2)	0.061
Hypoechoic	500 (67.5)	283 (67.2)	0.948
Markedly hypoechoic	62 (8.4)	15 (3.6)	0.001
Echogenic foci			
Microcalcifications	222 (30.0)	126 (29.9)	0.991
Macrocalcifications	117 (15.8)	61 (14.5)	0.611
Peripheral calcifications	33 (4.5)	9 (2.1)	0.049
Comet-tail artifacts	20 (2.7)	15 (3.6)	0.475
Outcome			0.903
Benign	365 (49.3)	209 (49.6)	
Malignant	376 (50.7)	212 (50.4)	

**Table 2:** Distribution of ultrasound features among malignant thyroid nodules.

Characteristics	Primary cohort n=376 (%)	Validation cohort n=212 (%)	*P*
Sex			0.915
Male	58 (15.4)	32 (15.1)	
Female	318 (84.6)	180 (84.9)	
Age (years)	48 (39-55)	44 (31.25-56.75)	0.008
Orientation			0.009
Parallel	118 (31.4)	45 (21.2)	
Vertical	258 (68.6)	167 (78.8)	
Margin			
Circumscribed	194 (51.6)	82 (38.7)	0.003
Ill-defined or irregular	151 (40.2)	122 (57.5)	< 0.001
Extrathyroidal extension	60 (16.0)	15 (7.1)	0.002
Composition			
Solid	372 (98.9)	205 (96.7)	0.064
Predominantly solid	4 (1.1)	7 (3.3)	0.064
Predominantly cystic	0 (0.0)	0 (0.0)	–
Echogenicity			
Iso/hyperechoic	12 (3.2)	7 (3.3)	0.942
Hypoechoic	315 (83.8)	190 (89.6)	0.064
Markedly hypoechoic	49 (13.0)	15 (7.1)	0.027
Echogenic foci			
Microcalcifications	175 (46.5)	95 (44.8)	0.730
Macrocalcifications	64 (17.0)	31 (14.6)	0.485
Peripheral calcifications	15 (4.0)	1 (0.5)	0.014
Comet-tail artifacts	0 (0.0)	0 (0.0)	–

### 3.2 Feature contribution and modified C-TIRADS scoring system

**Figure 2:**
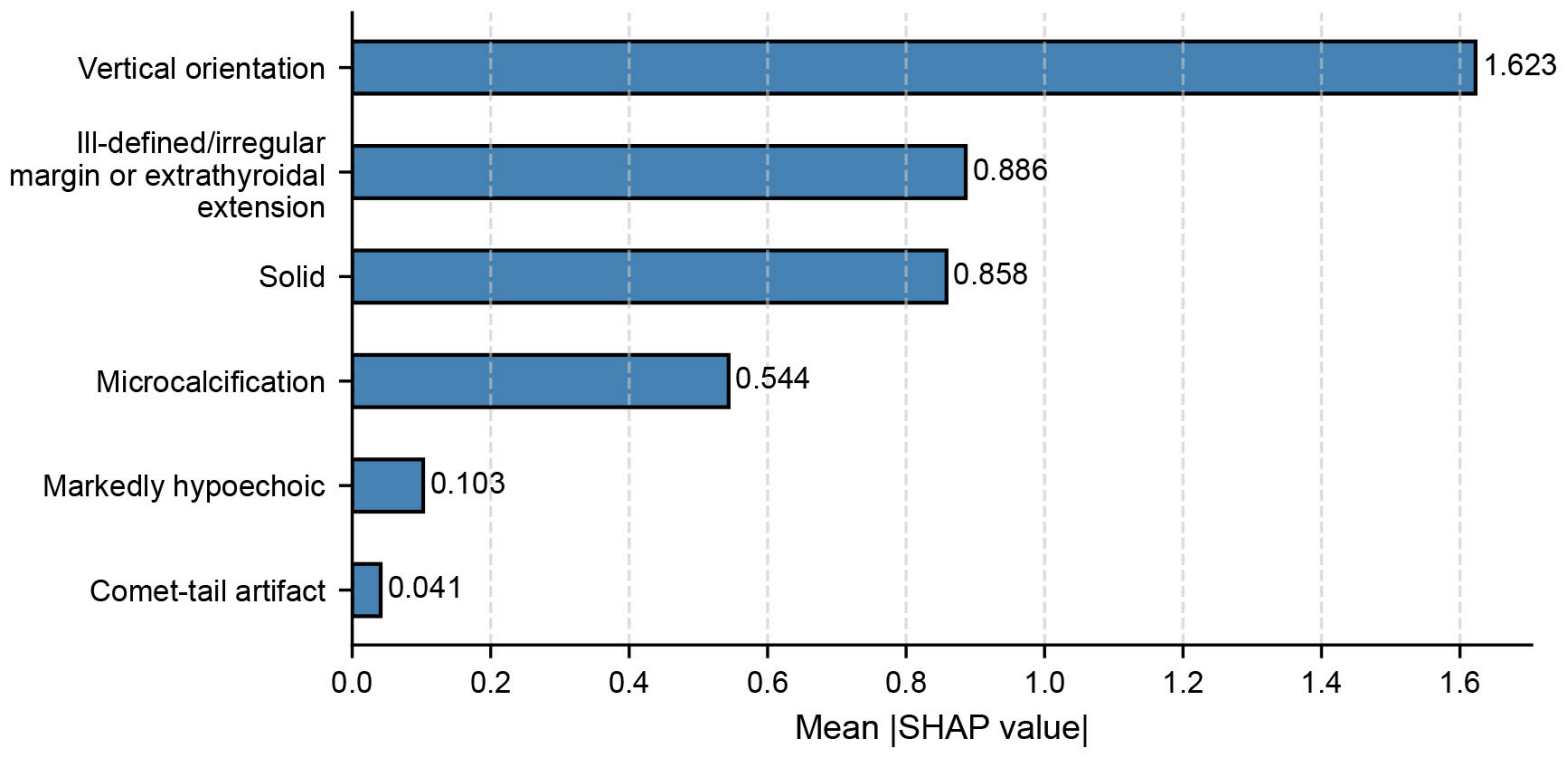
SHAP summary plot showing the relative contribution of ultrasound features to malignancy prediction in sub-centimeter nodules using XGBoost in the primary cohort.

SHAP analysis based on the XGBoost model revealed that vertical orientation was the most influential feature contributing to malignancy prediction in sub-centimeter nodules, followed by ill-defined/irregular margin or extrathyroidal extension, and solid composition (**Figure 2**). These findings informed the development of a modified C-TIRADS scoring system, in which vertical orientation was assigned 2 points. All other features retained their original scoring weights according to the 2020 C-TIRADS guideline.

### 3.3 Diagnostic performance comparison

**Figure 3:**
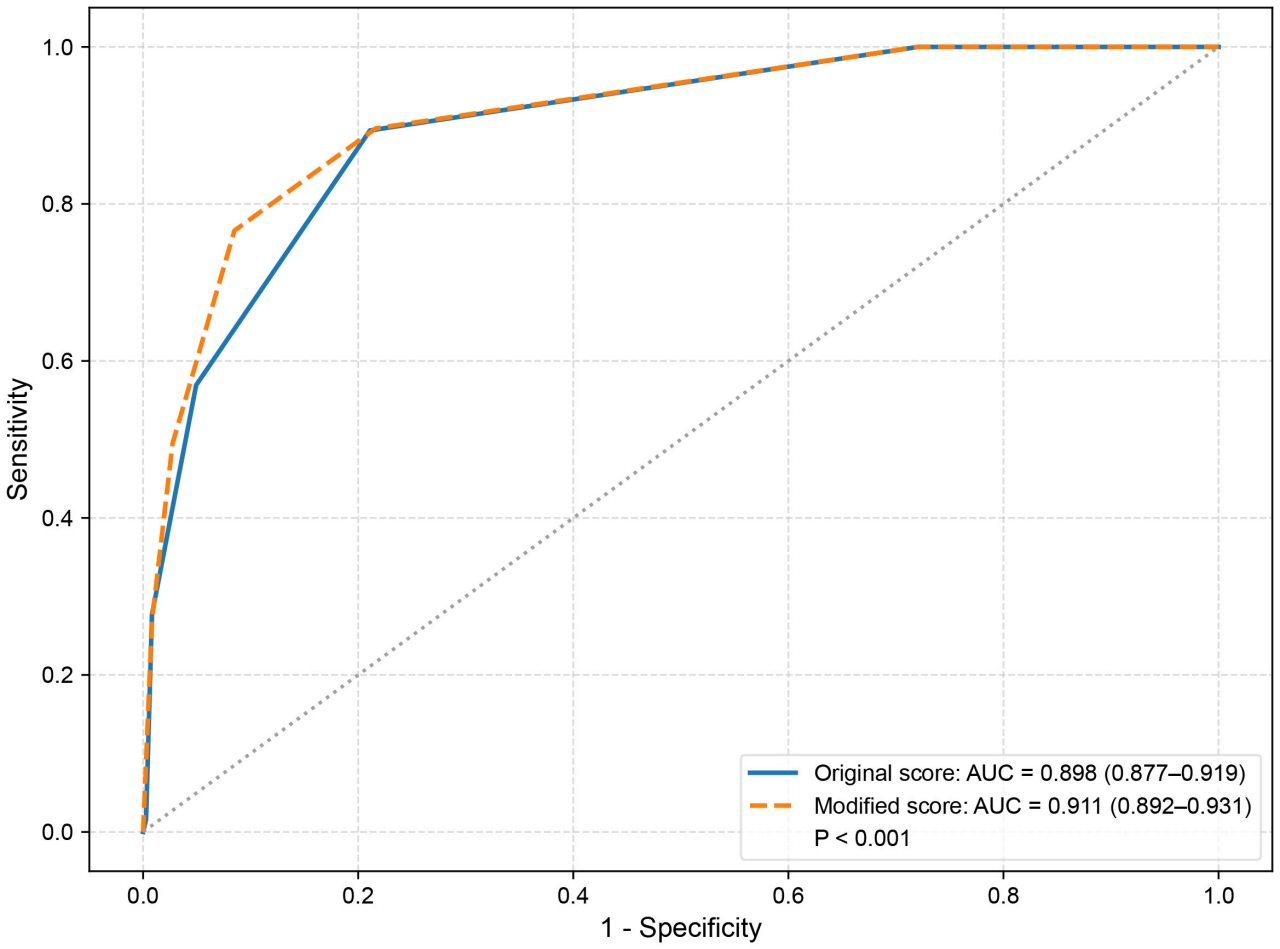
Receiver operating characteristic curves comparing diagnostic performance of the original and modified C-TIRADS scoring systems in the primary cohort.

ROC curve analysis demonstrated that the modified C-TIRADS scoring system had significantly better diagnostic performance compared with the original system in the primary cohort (**Figure 3**), with the AUC increasing from 0.898 to 0.911 (*P* < 0.001).

### 3.4 Net reclassification and risk migration

**Figure 4:**
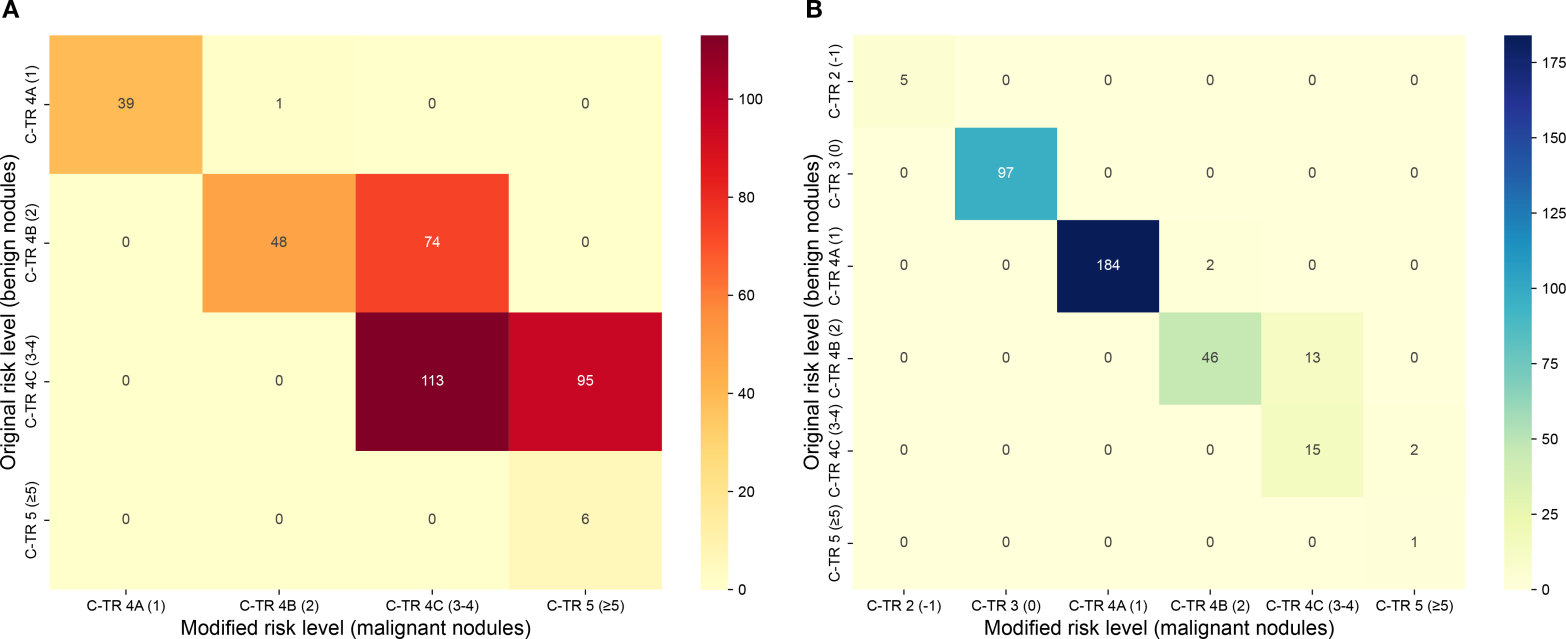
Heatmaps showing reclassification of benign and malignant nodules across C-TIRADS risk categories between the original and modified scoring systems in the primary cohort. **(A)** malignant nodules; **(B)** benign nodules.

NRI analysis showed a significant enhancement in risk classification with the modified scoring system. In the primary cohort, NRI was 0.406 (95% CI: 0.349–0.462, *P* < 0.001). Heatmaps in **Figure 4** illustrate the distributional changes between original and modified scoring categories for both benign and malignant nodules. The modified scoring system had a substantial increase in the upward reclassification of malignant nodules, especially in C-TIRADS category 4B (60.7%) and category 5 (45.7%). While a modest proportion of benign nodules were also misclassified into higher-risk levels in C-TIRADS 4B (22.0%) and C-TIRADS 4C (11.8%) categories.

**Table 3** summarizes the malignancy rates across TR categories defined by the original and modified C-TIRADS scoring systems. The modified system provided a clearer stratification trend, with a significantly higher malignancy rate in C-TIRADS 5 and lower rates in C-TIRADS 4A-4C categories compared to the original model (*P* < 0.001).

**Table 3:** Malignancy rates across TIRADS risk categories defined by original and modified C-TIRADS scoring systems in the primary and validation cohorts.

Risk levels	Original scoring (n, %)	Modified scoring (n, %)	*P*
Primary cohort			
C-TIRADS 4A	40 (17.7%)	39 (17.5%)	< 0.001
C-TIRADS 4B	122 (67.4%)	49 (50.5%)	
C-TIRADS 4C	208 (92.4%)	187 (87.0%)	
C-TIRADS 5	6 (85.7%)	101 (97.1%)	
Validation cohort			
C-TIRADS 4A	22 (19.3%)	15 (14.3%)	< 0.001
C-TIRADS 4B	60 (56.6%)	30 (41.1%)	
C-TIRADS 4C	122 (93.1%)	95 (88%)	
C-TIRADS 5	8 (100%)	72 (98.6%)	

### 3.5 Clinical benefit evaluation

**Figure 5:**
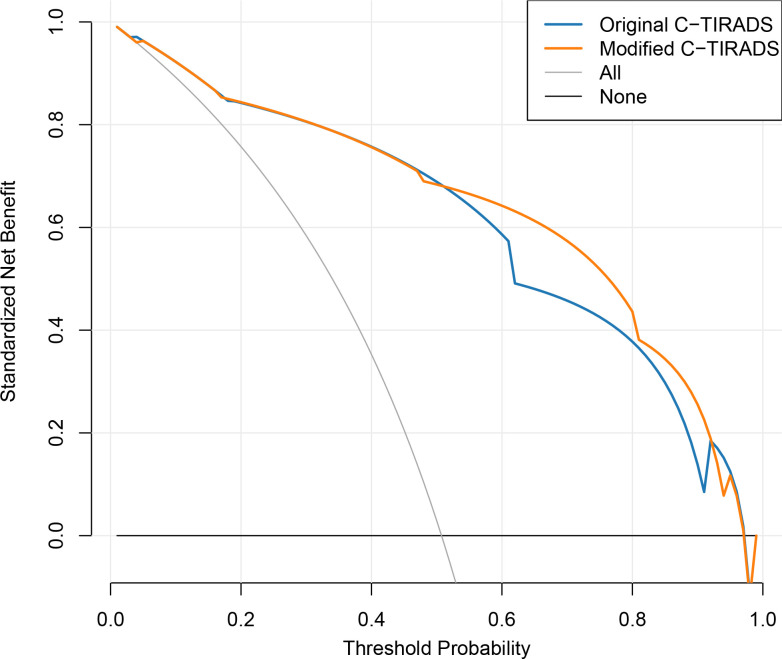
Decision curve analysis comparing net clinical benefit of the original and modified C-TIRADS scoring systems in the primary cohort.

As depicted in **Figure 5**, DCA demonstrated that the modified C-TIRADS scoring system offered greater net clinical benefit than the original system across threshold probabilities ranging from 52% to 92% in the primary cohort.

### 3.6 Validation of the modified scoring system

**Figure 6:**
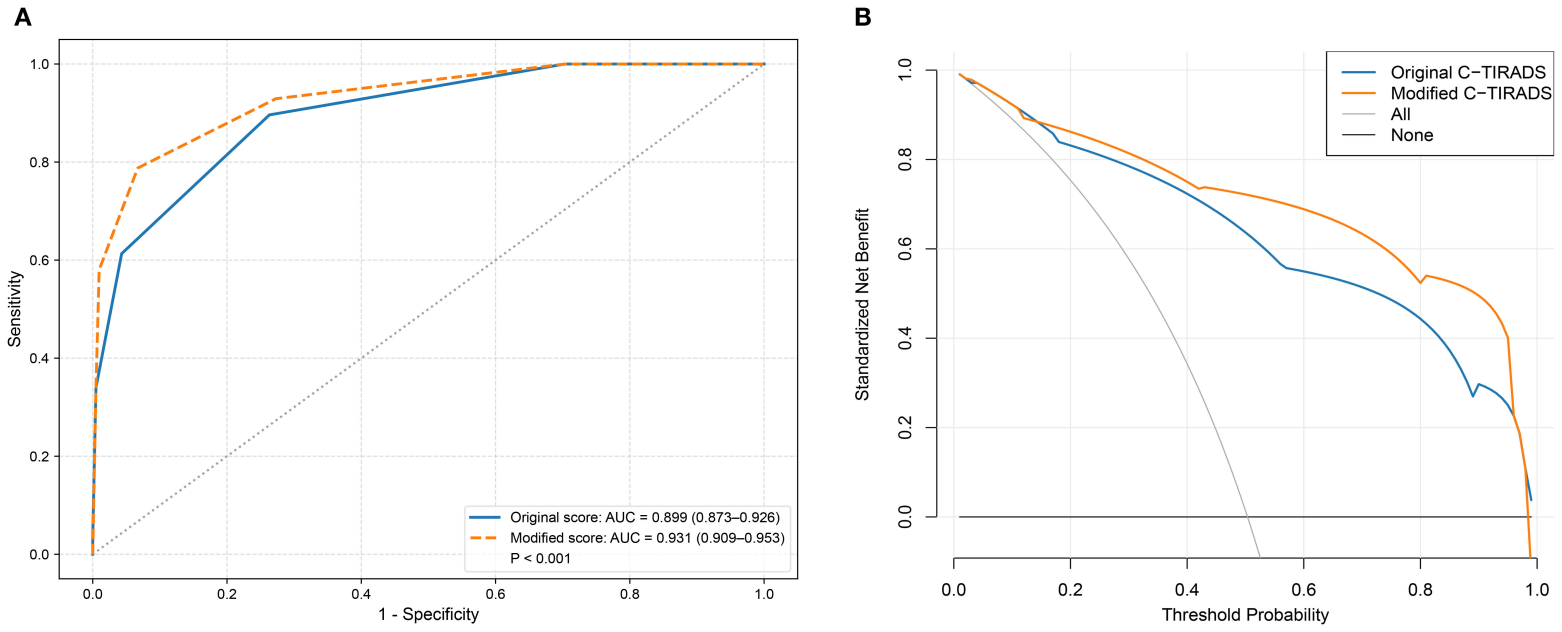
Receiver operating characteristic curves and decision curve analysis comparing the diagnostic performance and clinical net benefit of the original and modified C-TIRADS scoring systems in the validation cohort. **(A)** receiver operating characteristic curves; **(B)** decision curve analysis.

In the validation cohort, as shown in **Figure 6**, the modified C-TIRADS scoring system demonstrated superior diagnostic performance compared to the original version, with the AUC increasing from 0.899 to 0.931 (*P* < 0.001). NRI analysis indicated a significant improvement in risk stratification, with an NRI of 0.471 (95% CI: 0.400–0.542, *P* < 0.001), further supporting the enhanced discriminatory capacity of the modified model. Additionally, DCA (**Figure 6**) showed that the modified model yielded higher net clinical benefit across a broader threshold probability range (15% to 95%) compared with the original system.

Moreover, the modified scoring system exhibited improved risk stratification, characterized by a more rational stepwise increase in malignancy rates across C-TIRADS 4A to 5 and a more appropriate allocation of malignant nodules to higher categories (*P* < 0.001, **Table 3**). The validation results corroborated the findings from the primary cohort, lending further support to the effectiveness of the modified C-TIRADS system.

## 4 Discussion

In this study, we developed and validated a size-specific modification of the C-TIRADS scoring system for sub-centimeter thyroid nodules, using SHAP-informed feature weighting derived from a machine learning model. Our findings demonstrated that the modified scoring system, which assigned greater weight to vertical orientation, the most predictive feature in small nodules, achieved superior diagnostic performance, improved malignancy risk stratification, and enhanced clinical utility compared with the original C-TIRADS guideline. These results were consistently observed in both the primary and external validation cohorts, supporting the robustness and generalizability of the modified system.

Although most international guidelines, including those from the American Thyroid Association, recommend active surveillance or conservative management for nodules ≤1 cm, a growing body of evidence suggested (13, 14) that a small but significant proportion of cases harbor aggressive histological features, such as extrathyroidal extension, lymph node metastasis, and BRAF mutations, even at early stage. A meta-analysis reported that the overall incidence of central lymph node metastases in papillary thyroid microcarcinoma patients was 33% (15). Improving diagnostic performance for sub-centimeter thyroid nodules is therefore clinically important, yet remains challenging. Due to their small size and frequently ambiguous sonographic appearance, these nodules are more likely to be underdiagnosed or misclassified by conventional scoring systems. This diagnostic uncertainty may lead to missed malignancies or delayed interventions in patients with clinically significant microcarcinomas. Hence, a size-tailored and more accurate risk stratification approach, the SHAP-informed modified C-TIRADS proposed in this study, is essential to better identify microcarcinomas, thereby supporting more personalized and effective clinical decision-making.

In our SHAP analysis, vertical orientation emerged as the most important predictor of malignancy among the six C-TIRADS features in sub-centimeter nodules, consistent with previous findings (12). Thyroid microcarcinomas more frequently exhibit a “taller-than-wide” configuration, in which the anteroposterior dimension exceeds the transverse dimension. The majority of microcarcinomas tend to arise in a subcapsular or peripheral location, which predisposes the tumor to invade outward, following the path of least resistance, into the adjacent soft tissue structures rather than expanding laterally within the confined parenchyma of the thyroid (16). Additionally, the tall cell variant of papillary thyroid carcinoma, defined as tumor cells at least twice as tall as they are wide, is associated with a more aggressive phenotype (17), which may contribute to vertical orientation. Moreover, the hobnail variant of thyroid carcinoma is a distinctive pattern whereby tumor cells loss of cellular polarity/cohesiveness (18). In these tumors, cells lose their normal orientation and cell-to-cell adhesion is diminished, which may result in a vertical growth behavior. These morphological and histopathological patterns provide a biological rationale for assigning increased weight to vertical orientation in the modified scoring system.

Furthermore, the modified scoring system exhibited superior risk stratification, with progressively increasing malignancy rates across C-TIRADS categories 4A to 5 and a more appropriate allocation of malignant nodules to higher-risk categories. This modified model improves the evaluation and stratification of high-risk sub-centimeter nodules and is helpful for clinical decision-making, especially regarding whether to perform fine-needle aspiration or adopt active surveillance.

It is noteworthy that recent studies have explored the potential value of incorporating additional ultrasound features or clinical parameters into risk stratification models. For example, one study (19) demonstrated that combining TIRADS scoring systems with thyroid-stimulating hormone could improve the sensitivity of predicting differentiated thyroid carcinoma, and other studies (20–22) have shown that vascularity, elastography, and contrast-enhanced ultrasound may further enhance the diagnostic accuracy of TIRADS. However, C-TIRADS has been widely adopted in China due to its simplicity and reasonable diagnostic performance. To maintain its ease of use, our study focused solely on optimizing the six intrinsic features defined by C-TIRADS, without incorporating additional factors, although integrating further ultrasound features or clinical parameters may further improve the diagnostic performance of risk stratification models. In future studies, integrating additional features and developing user-friendly tools such as nomograms or online calculators may further facilitate the clinical application of the modified C-TIRADS system. Additionally, our findings suggest that the modified C-TIRADS system may have potential implications for refining fine-needle aspiration decision-making in sub-centimeter nodules. This warrants further investigation in future research.

Despite these promising results, several limitations should be acknowledged. First, this was a retrospective study and may be subject to inherent selection bias. Second, although the external validation cohort supported the modified model, both cohorts were derived from tertiary medical centers within the same city. This geographic and institutional homogeneity may limit the generalizability of our findings to other populations and practice settings. Therefore, our results should be interpreted as preliminary evidence, and further multicenter validation is warranted to confirm the robustness and applicability of the modified C-TIRADS system. Third, this study specifically focused on the six ultrasound features explicitly defined in the 2020 C-TIRADS guideline, since the primary objective was to optimize the C-TIRADS scoring system itself. Thus, additional features such as vascularity, elastography, contrast-enhanced ultrasound, thyroid-stimulating hormone, or BRAF mutation were not included. Nevertheless, incorporating these features may further enhance diagnostic accuracy and should be explored in future prospective studies with standardized protocols. Fourth, information on overall thyroid size, multinodular goiter, Hashimoto thyroiditis, and Graves disease was not systematically collected in this retrospective study. Therefore, we were unable to evaluate the potential impact of these conditions on the association between vertical orientation and malignancy. Finally, although SHAP analysis provides model explainability, prospective validation remains necessary for widespread implementation.

## 5 Conclusions

In conclusion, this study proposed a SHAP-guided modification of the C-TIRADS scoring system tailored for sub-centimeter thyroid nodules. By assigning greater weight to vertical orientation, the modified scoring system achieved better diagnostic performance and more accurate risk stratification for sub-centimeter nodules, facilitating the identification of high-risk sub-centimeter nodules in clinical practice.

## Data Availability

The data analyzed in this study is subject to the following licenses/restrictions: The datasets of this study are available from the corresponding author upon reasonable request. Requests to access these datasets should be directed to Zhe Chen, zchensdzl@163.com.
