# Correction: Optimizing C-TIRADS for sub-centimeter thyroid nodules using machine learning–derived feature importance

**DOI:** 10.3389/fendo.2025.1718347

**Published:** 2025-10-31

**Authors:** Dongming Guo, Zhihui Lin, Jiajia Wang, Xianying Liao, Haiqing Huang, Yuxia Zhai, Zhe Chen

**Affiliations:** ^1^ Department of Interventional Ultrasound, Cancer Hospital of Shantou University Medical College, Shantou, China; ^2^ Department of Ultrasound, Cancer Hospital of Shantou University Medical College, Shantou, China; ^3^ Department of Ultrasound, Second Affiliated Hospital of Shantou University Medical College, Shantou, China

**Keywords:** sub-centimeter thyroid nodules, C-TIRADS, machine learning, SHAP, ultrasound, risk stratification, microcarcinoma

**Figures 4** and **5** were in the wrong order in the PDF and HTML versions of this paper. **Figures 4** and **5** were mistakenly swapped. **Figure 4** should display the risk stratification heatmap and **Figure 5** should display the Decision Curve Analysis. The order has now been corrected.

**Figure 4:**
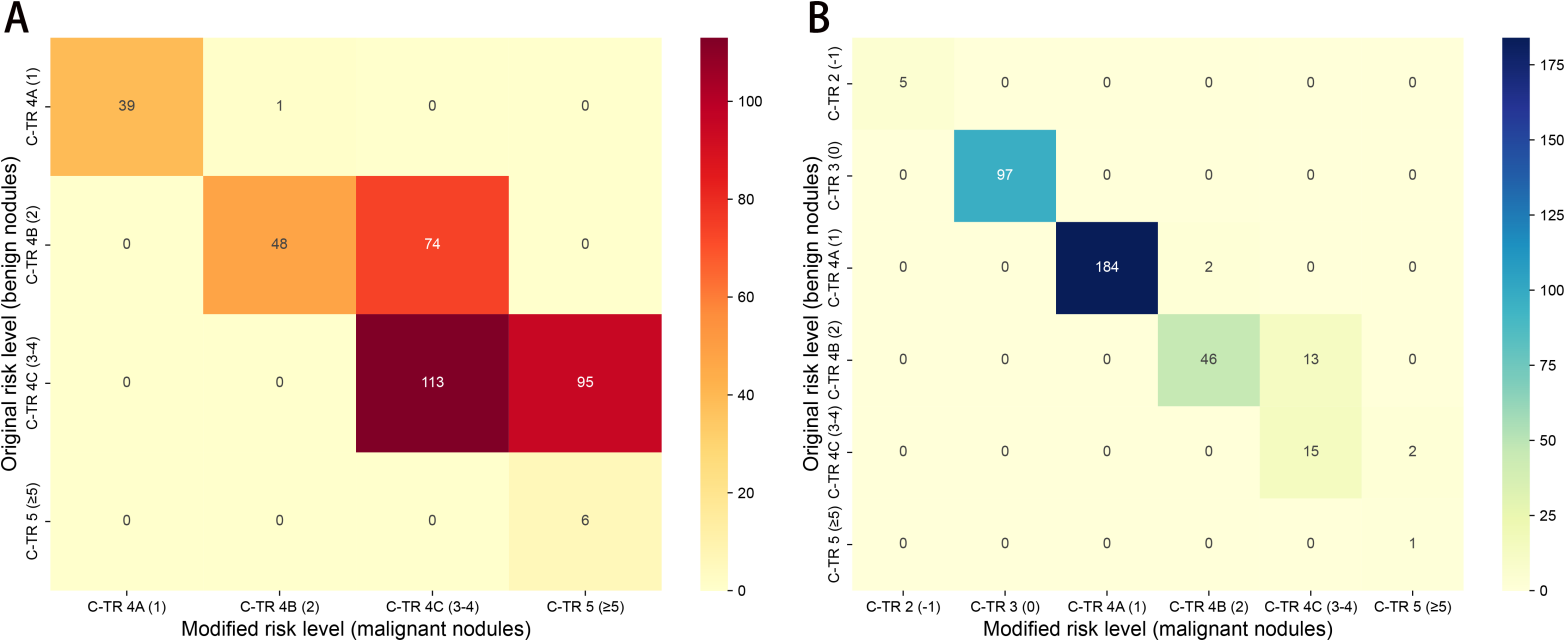
Heatmaps showing reclassification of benign and malignant nodules across C-TIRADS risk categories between the original and modified scoring systems in the primary cohort. **(A)** malignant nodules; **(B)** benign nodules.

**Figure 5:**
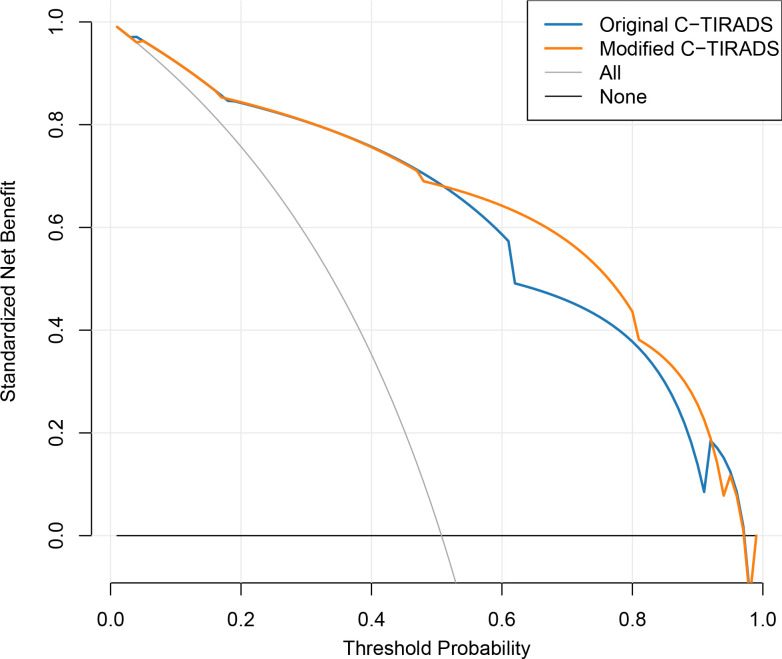
Decision curve analysis comparing net clinical benefit of the original and modified C-TIRADS scoring systems in the primary cohort.

The original version of this article has been updated.

